# Early detection of central line-associated bloodstream infection in intensive care unit patients using the systemic inflammatory response index (SIRI)

**DOI:** 10.3205/dgkh000606

**Published:** 2025-12-05

**Authors:** Rijhul Lahariya, Gargee Anand

**Affiliations:** 1 Department of Microbiology, All India Institute of Medical Sciences, Patna, Bihar, India

**Keywords:** systemic inflammatory response index, SIRI, central line-associated bloodstream infection, CLABSI, inflammatory biomarkers

## Abstract

**Objective:**

Central line-associated bloodstream infections (CLABSIs) are life-threatening complications in critically ill patients, necessitating early identification for timely intervention. This study evaluates the predictive performance of Systemic Inflammatory Response Index (SIRI), a novel composite marker derived from routine blood counts, for early prediction of CLABSI within first two calendar days following central venous catheter (CVC) insertion.

**Method:**

In this observational study at a tertiary ICU, 234 adults with CVCs for over two days were classified as CLABSI-positive or negative per CDC/NHSN criteria. SIRI was calculated using the formula (neutrophils×monocytes/lymphocytes) based on day 2 complete blood counts. Logistic regression and receiver operating characteristic (ROC) curve analysis was done to determine diagnostic performance.

**Results:**

CLABSI was diagnosed in 39 patients. Median SIRI values were significantly higher in CLABSI group (37.3 vs. 12.0; p<0.001). In univariate logistic regression, SIRI emerged as an independent predictor of CLABSI (OR=1.0097; 95% CI: 1.001–1.018; p=0.015). ROC analysis demonstrated a moderate discriminative power with AUROC=0.72 (95% CI: 0.64–0.80). At optimal threshold, SIRI achieved 84.6% sensitivity, 52.8% specificity, 58.1% accuracy, 26.4% positive predictive value (PPV) and a notably high negative predictive value (NPV) of 94.5%, supporting its value as an early rule-out marker for CLABSI.

**Conclusion:**

SIRI, derived from routine complete blood counts, shows strong potential as a non-invasive, early screening marker for CLABSI. Its high sensitivity and NPV support its use for early rule-out, especially in settings lacking rapid diagnostics. Further prospective validation is warranted.

## Zusammenfassung

**Zielsetzung:**

Zentralvenenkatheter-assoziierte Blutstrominfektionen (CLABSI) sind lebensbedrohliche Komplikationen bei kritisch kranken Patienten, die eine frühzeitige Erkennung erfordern, um rechtzeitig eingreifen zu können. Die Studie bewertet die Vorhersagekraft des systemischen inflammatorischen Response Indexes (SIRI), eines neuartigen kombinierten Markers, der für die frühzeitige Vorhersage von CLABSI aus routinemäßigen Blutbildwerten innerhalb der ersten zwei Kalendertage nach der Einlage eines zentralvenösen Katheters (CVC) abgeleitet wird.

**Methode:**

In der Beobachtungsstudie auf der Intensivtherapiestation eines Krankenhauses der Tertiärversorgung wurden 234 Erwachsene mit CVCs, die länger als zwei Tage angelegt waren, gemäß den CDC/NHSN-Kriterien als CLABSI-positiv oder -negativ eingestuft. Der SIRI wurde anhand der Formel (Neutrophile×Monozyten/Lymphozyten) auf der Grundlage der vollständigen Blutbilder am zweiten Tag berechnet. Zur Bestimmung der diagnostischen Leistungsfähigkeit wurden eine logistische Regression und eine Receiver Operating Characteristic (ROC)-Kurvenanalyse durchgeführt.

**Ergebnisse:**

Bei 39 Patienten wurde eine CLABSI diagnostiziert. Die medianen SIRI-Werte waren in der CLABSI-Gruppe signifikant höher (37,3 vs. 12,0; p<0,001). In der univariaten logistischen Regression erwies sich SIRI als unabhängiger Prädiktor für CLABSI (OR=1,0097; 95% KI: 1,001–1,018; p=0,015). Die ROC-Analyse zeigte eine moderate Unterscheidungskraft mit AUROC=0,72 (95% KI: 0,64–0,80). Bei optimalem Schwellenwert erreichte SIRI eine Sensitivität von 84,6%, eine Spezifität von 52,8%, eine Genauigkeit von 58,1%, einen positiven prädiktiven Wert (PPV) von 26,4% und einen bemerkenswert hohen negativen prädiktiven Wert (NPV) von 94,5%, was seinen Wert als Marker für die frühzeitige Ausschließung von CLABSI untermauert.

**Schlussfolgerung:**

SIRI, abgeleitet aus routinemäßigen vollständigen Blutbildern, zeigt ein starkes Potenzial als nicht-invasiver Marker für die Früherkennung von CLABSI. Seine hohe Sensitivität und sein hoher NPV sprechen für seine Verwendung zur frühzeitigen Ausschlussdiagnostik, insbesondere wenn keine Schnelldiagnostik verfügbar ist. Eine weitere prospektive Validierung ist erforderlich.

## Introduction

Among HAIs, CLABSIs, as highlighted by recent Centers for Disease Control and Prevention (CDC) data remain a major clinical and public health concern due to their rapid progression, significant impact on patient outcomes and continued high burden across healthcare settings [1]. CLABSIs pose a serious threat in critical care, driving longer intensive care unit (ICU) stays, higher morbidity and mortality and escalating healthcare costs worldwide [2], [3]. Their rapid progression to severe conditions like sepsis and multi-organ failure highlights the urgent need for early detection and prompt intervention to prevent poor outcomes [4]. Blood cultures remain the gold standard for diagnosing bloodstream infections (BSI), including CLABSI, but their clinical utility is often limited by delayed turnaround times, low sensitivity in early infection and frequent false positives from skin flora contamination [5]. These drawbacks can lead to unnecessary antibiotic use, delayed treatment and rising antimicrobial resistance [6]. This underscores a critical unmet need for rapid, reliable and cost-effective biomarkers to enable early detection and timely management of CLABSI, especially in high-risk ICU settings.

Systemic inflammatory markers derived from routine complete blood counts (CBC) are gaining attention as early red flags for infection [7]. While simple ratios like neutrophil-to-lymphocyte (NLR) and platelet-to-lymphocyte (PLR) have shown potential in infectious diseases, their role in the early detection of CLABSI remains underexplored – presenting a promising yet untapped opportunity for timely intervention in critical care [8], [9], [10], [11], [12]. 

Central venous catheter (CVC) insertion triggers more than just a local reaction – it sets off a systemic immune response [12], [13]. Endothelial injury at the catheter site activates an inflammatory cascade, leading to a surge in neutrophils driven by delayed cell death, a drop in lymphocytes due to immune suppression and a transient dip in monocytes as they migrate into tissues [14], [15], [16], [17], [18]. These dynamic shifts in immune cell profiles may offer early clues to impending BSIs, including CLABSI. 

These immune changes lay the foundation for novel composite markers like the Systemic Inflammatory Response Index (SIRI), which combines routine CBC parameters to capture the body’s inflammatory response. While SIRI has shown promise in predicting outcomes in sepsis, acute kidney injury and type 1 diabetes mellitus patients, its role in the early detection of CLABSI within 2 calendar days of central line insertion remains largely unexplored –highlighting a critical gap in current diagnostic strategies [19], [20], [21], [22]. 

Given that CBC is routinely performed within hours of ICU admission, this study aims to evaluate the predictive potential of the SIRI – a simple, cost-effective marker derived from CBC – for early prediction of CLABSI within the first two calendar days of central line insertion in critically ill adults. 

## Materials and methods

### Patient selection and data collection

 This retrospective observational study was conducted in the ICUs of a tertiary care hospital, targeting adult patients aged ≥18 years. A total sample size of 260 was determined using the Taro Yamane formula, ensuring a 95% confidence level with a 5% margin of error. Eligible participants included ICU patients with CVCs in place for more than two calendar days. Patients with documented BSI at the time of ICU admission were excluded from the analysis. Case identification and surveillance were performed in accordance with the standardized definitions outlined by the CDC National Healthcare Safety Network (NHSN) [23]. All data were fully anonymized before analysis to protect patient confidentiality. As this study did not involve the use of patient’s personal data, ethical approval was waived off. Also this was a retrospective study based on anonymized data, without any direct patient contact or use of identifiable personal information, informed consent was not applicable.

Key demographic variables (age and sex) and body temperature were recorded on the second calendar day following CVC insertion, along with routinely available blood-derived inflammatory indices. These variables were selected based on their established relevance to systemic inflammatory responses, consistent measurement reliability, and universal availability in clinical settings. The inclusion of only essential and readily accessible parameters enhances the feasibility and applicability of this approach, particularly in resource-constrained healthcare environments where comprehensive diagnostic resources may be limited. 

The calculation of inflammatory indices based on CBC included the following [24]: 

SIRI=neutrophils×monocytes/lymphocytes 

### Statistical analysis

All data were analysed using IBM SPSS Statistics version 26. The normality of continuous variables was assessed using the Shapiro-Wilk test. Continuous variables were expressed as mean±standard deviation (SD) or median with interquartile range (IQR), based on their distribution, while categorical variables were reported as frequencies and percentages. Patients were stratified into CLABSI-positive and CLABSI-negative groups. Intergroup comparisons were performed using the Independent t-test or Mann-Whitney U test for continuous variables, depending on distribution and the Chi-square test for categorical variables. Correlation between SIRI and continuous clinical parameters was determined using Pearson or Spearman rank correlation test, based on the data distribution. 

The diagnostic performance of SIRI for early CLABSI prediction was further assessed using area under the receiver operating characteristic (AUROC) curve, along with sensitivity, specificity, accuracy, positive predictive value (PPV) and negative predictive value (NPV). Odd’s ratio (OR) with its 95% confidence interval was calculated. ROC plots were generated and customized using Python version 3.10.12 with the ‘matplotlib’ library. A two-sided p-value ≤0.05 was considered statistically significant. 

## Results

A total of 234 adult ICU patients met the inclusion criteria and were enrolled in the study. Demographic as well as temperature did not differ significantly between CLABSI-positive and CLABSI-negative groups (Table 1 (Tab. 1)). 

Although demographic and clinical variables did not show significant differences between CLABSI-positive and CLABSI-negative groups, the SIRI demonstrated a notable distinction. By integrating neutrophil, monocyte and lymphocyte counts, SIRI captures early immune dysregulation more sensitively than individual leucocyte parameters alone. Prior to univariate logistic regression analysis, potential confounding effects of age and temperature were assessed using Spearman’s rank correlation. No significant associations were observed between SIRI and either variable, suggesting they did not confound the relationship between SIRI and CLABSI.

To evaluate the predictive utility of the SIRI for early CLABSI detection, a univariate binary logistic regression analysis was done with the CLABSI-negative group as reference. SIRI was found to be a statistically significant predictor of CLABSI, with an OR of 1.0097 (95% CI: 1.001–1.018; p-value=0.015). This indicates that a 10 unit increase in SIRI was associated with 10% higher odds of CLABSI. The p-value was <0.05, indicating that higher value of SIRI was linked to a greater likelihood of developing CLABSI. 

**Figure 1:**
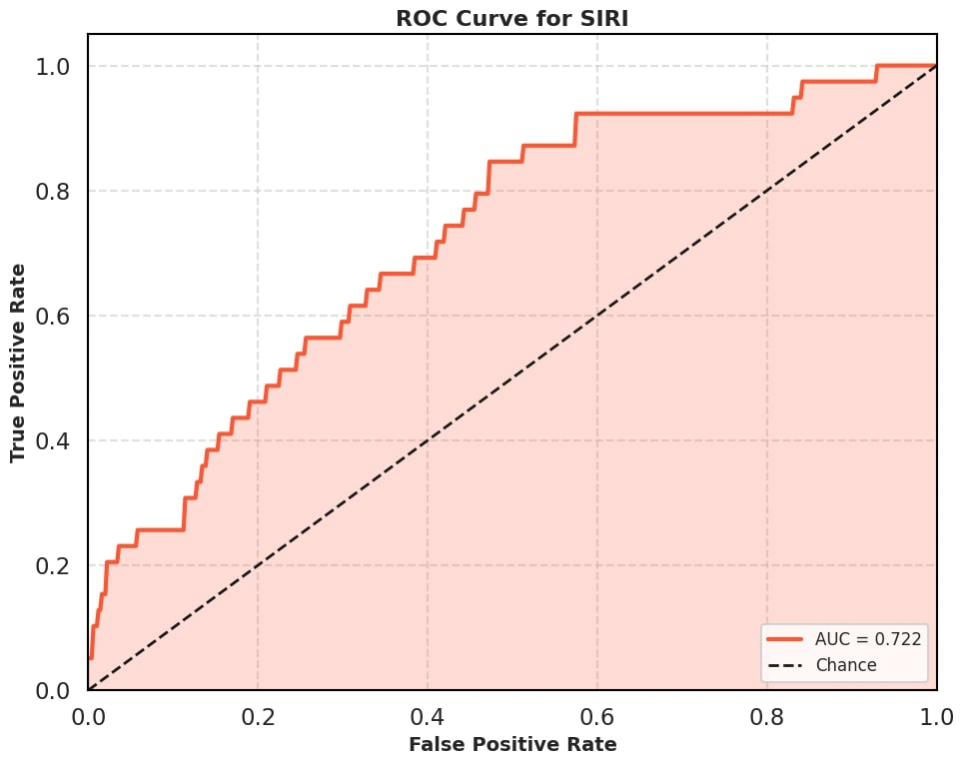
Receiver operating characteristic curve to look for the diagnostic performance of SIRI for predicting CLABSI.

The diagnostic performance of SIRI was further assessed using ROC curve analysis. SIRI demonstrated an AUROC of 0.72 (95% CI: 0.64–0.8), suggesting moderate discriminative ability (Figure 1 (Fig. 1)). 

At the optimal cutoff, SIRI achieved a sensitivity of 84.6%, specificity of 52.8%, and a high NPV of 94.5%, indicating strong utility as a rule-out tool for early CLABSI detection (Table 2 (Tab. 2)). These findings underscore the potential of SIRI as a simple, cost-effective inflammatory marker to support early clinical decision-making in critically ill patients with CVCs. 

## Discussion

CLABSIs continue to be a significant contributor to mortality and extended ICU stays, underscored by unprecedented multidrug resistance that has rendered most therapies ineffective, highlighting the urgent need for reliable methods for early detection [24], [25]. Conventional diagnostic methods, based on clinical suspicion and standard blood tests, often fail to capture subtle immune shifts during the early stages of infection, delaying timely intervention [5]. This study investigates the novel use of a composite inflammatory index SIRI, which combine neutrophil, leucocyte and monocytes value to improve early infection detection. Notably, individual leucocyte, monocyte and neutrophil counts did not show significant differences between the CLABSI-positive and negative groups, underscoring the added value of these integrated indices. 

SIRI emerged as a significant independent predictor of CLABSI in logistic regression analysis (p-value=0.015). It demonstrated strong diagnostic performance with an AUROC of 0.72, high sensitivity (84.6%), and acceptable specificity (52.8%). These findings highlight SIRI's potential as a simple, cost-effective tool for early identification of CLABSI risk, enabling timely clinical intervention before microbiological confirmation.

Following CVC insertion, the mechanical disruption of the endothelial lining initiates a localized inflammatory response, creating a vulnerable entry point for potential infection [26]. This breach facilitates microbial adhesion and biofilm formation on the catheter surface, a key step in the pathogenesis of CLABSI [27], [28]. In response, the innate immune system rapidly activates: neutrophils are the first to arrive, releasing reactive oxygen species (ROS), proteolytic enzymes and forming neutrophil extracellular traps to contain the invading pathogens [29]. Monocytes are subsequently recruited and differentiate into macrophages, which amplify the inflammatory response by secreting cytokines such as IL-6 and TNF-α [30]. Endothelial cells upregulate adhesion molecules, enhancing leucocyte recruitment to the site of injury [31]. Simultaneously, lymphocyte levels may decline transiently, reflecting redistribution or stress-induced suppression, further influencing the host’s capacity to mount an effective immune defense [32]. Together, these cellular changes lay the foundation for systemic inflammation and potential BSI if unchecked.

Recent evidence has highlighted the potential of this inflammatory index in identifying BSIs and systemic inflammation. Notably, elevated SIRI levels have been associated with catheter-related bloodstream infections (CRBSIs) in hemodialysis patients, showing promising diagnostic accuracy [33]. Consistent with these findings, our study demonstrated that higher SIRI values were significantly linked to CLABSI. With a sensitivity of 84.6% and a specificity of 52.8%, SIRI showed robust performance as an early, non-invasive screening tool. These findings underscore SIRI’s clinical utility in facilitating early CLABSI detection, especially in settings where timely microbiological confirmation may be delayed.

SIRI emerges as a promising early warning tool for CLABSI, especially in resource-constrained settings where rapid diagnostics are limited. With its high sensitivity, SIRI helps clinicians confidently rule out infection in the crucial early window, enabling faster decision-making and targeted care. By combining neutrophil, monocyte and lymphocyte counts into a single, powerful index, SIRI captures the body’s inflammatory response more holistically – offering a practical, cost-effective edge in infection surveillance [34]. We only looked at CBC results from the first two calendar days after insertion, so using this marker beyond this period would require further study with repeated measurements. 

Our study is the first, to our knowledge, to evaluate the predictive utility of the SIRI within the first two calendar days following central line insertion for early CLABSI detection. This critical time window is essential for initiating timely interventions before microbiological confirmation. SIRI, a cost-effective and easily derived index from routine blood counts, showed promising potential as an early screening tool for timely triage. However, certain limitations must be acknowledged. The retrospective design may introduce selection bias, potentially influencing the observed associations. Additionally, our relatively small sample size – stemming from strict eligibility criteria and a low CLABSI incidence at our center – may limit the generalizability of the findings.

## Conclusion

This study underscores the promising role of the SIRI, derived from routine CBC, as an early, non-invasive screening marker for CLABSI. Within the first two calendar days post–central line insertion, elevated SIRI levels demonstrated strong predictive performance, with high sensitivity and a robust negative predictive value – making it a valuable tool for early risk stratification. Given its simplicity, low cost and seamless integration into existing ICU workflows, SIRI offers particular clinical value in resource-limited settings where timely microbiological confirmation may not be feasible. However, these findings are based on a single-center cohort with a limited sample size. Larger prospective studies are warranted to validate SIRI’s diagnostic utility and establish standardized thresholds for clinical use. If validated, SIRI could enhance early CLABSI detection and inform timely preventive strategies in critically ill patients.

## Notes

### Authors’ ORCIDs

- Lahariya R: https://orcid.org/0009-0003-5769-4509
- Anand G: https://orcid.org/0009-0008-0473-389X

### Funding

None. 

### Acknowledgments

The authors acknowledge all consultants in the Department of Microbiology for their guidance and assistance.

### Competing interests

The authors declare that they have no competing interests.

## Figures and Tables

**Table 1:**
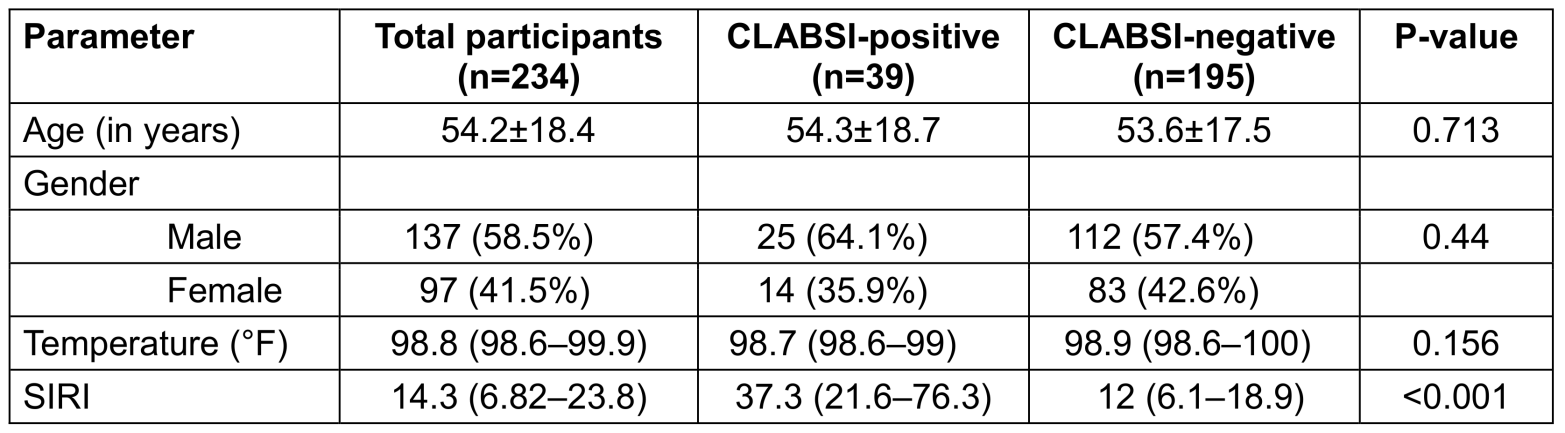
Demographic, clinical and inflammatory indices among CLABSI-positive and -negative patients

**Table 2:**
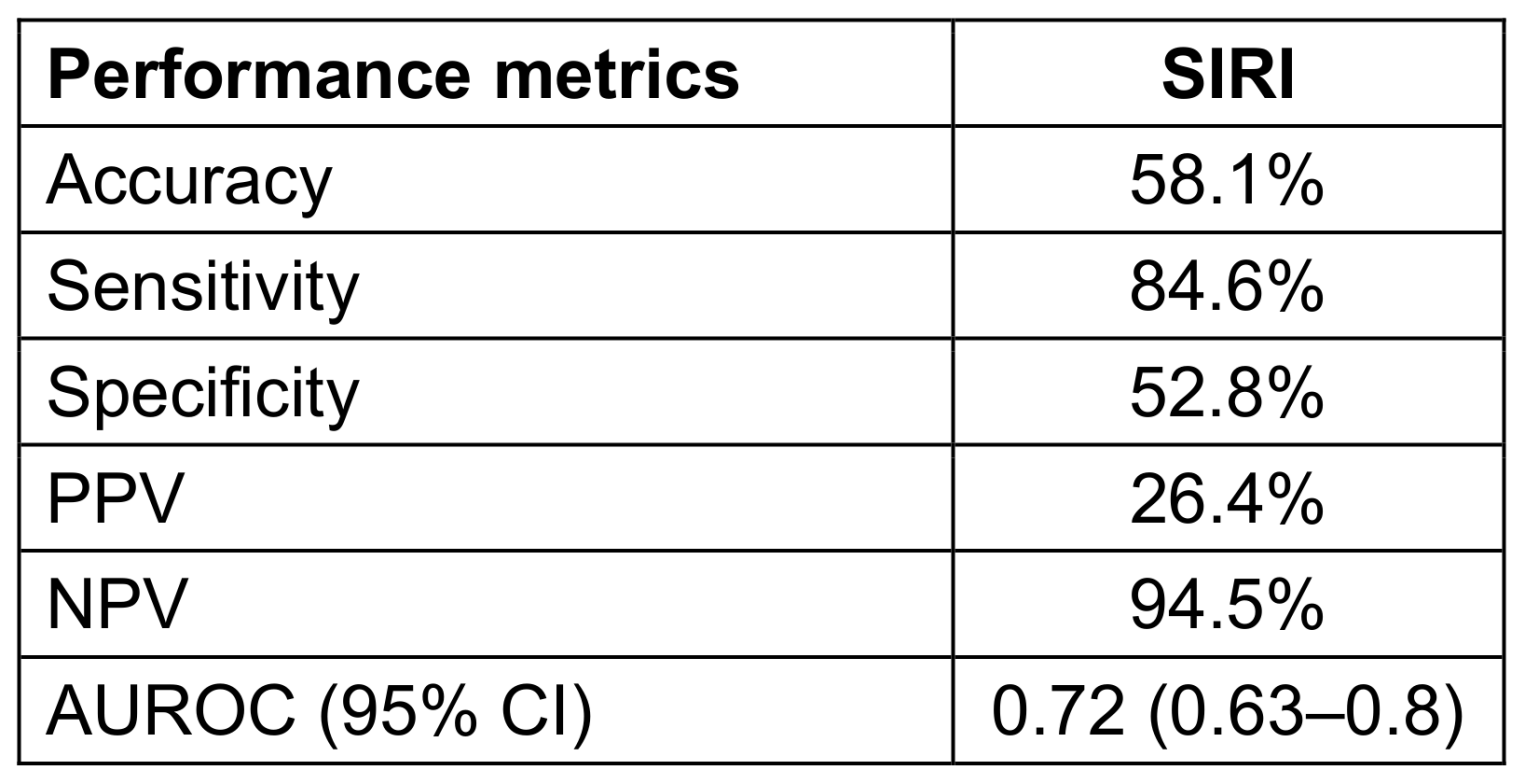
Odd’s ratio and performance metrics of SIRI in predicting CLABSI using logistic regression analysis.
